# Incidental morphological findings in colorectal adenomas

**DOI:** 10.1111/his.14263

**Published:** 2020-11-17

**Authors:** Parag D Dabir, Rachel S van der Post, Iris D Nagtegaal

**Affiliations:** ^1^ Department of Pathology Radboud University Medical Centre Nijmegen The Netherlands; ^2^ Institute of Pathology Randers Regional Hospital Randers Denmark

**Keywords:** clear cell metaplasia, colorectal adenomas, neuroendocrine differentiation, osseous metaplasia, Paneth cell metaplasia, population screening, signet‐ring cell‐like, squamous metaplasia

## Abstract

Owing to a sharp increase in the frequency of diagnosis of colorectal adenomas in the current era of population screening, distinctive morphological features are increasingly being observed. These may present diagnostic challenges and cause clinical management issues. Paneth cell metaplasia is a more common occurrence, but the incidence rates of squamous metaplasia, clear cell metaplasia, osseous metaplasia, neuroendocrine differentiation and signet‐ring cell‐like lesion are low, and they can be seen in <1% of colorectal adenomas. Their histomorphological characteristics are quite unique; ancillary studies are not very helpful and often not needed. In this review, we give an overview and describe the potential clinical consequences of such incidental and special morphological findings in colorectal adenomas.

## Introduction

High numbers of colorectal adenomas are currently being diagnosed, owing to the introduction of population screening programmes for colorectal cancer (CRC).^1^ These programmes are aimed at early detection of CRC, but also at the prevention of CRC by removing the precursor lesions. The majority of these precursors are conventional adenomas^2^: tubular, tubulovillous and villous adenomas, with either low‐grade or high‐grade dysplasia. Adenomas with distinct morphologies are increasingly being encountered, and are characterised by the presence of special cell types, incidental findings, or metaplasias.^2^, ^3^

These variants may lead to diagnostic difficulties. For example, the presence of squamous metaplasia,^4^ neuroendocrine differentiation^5^ or signet‐ring cell‐like lesions^6^ could give a false indication of invasive cancer. Although these variants are rare, awareness of them among pathologists and clinicians is important, especially in the current era.

On the basis of incidence rate, we give an overview of the morphological characteristics, biological background and clinical implications of these special and incidental findings in conventional adenomas. The clinical implications are defined as relevant during the diagnostic work‐up (i.e. this feature might be a diagnostic pitfall) or relevant for clinical management (i.e. increased surveillance because of high‐risk features). If there are no clinical implications mentioned, this means that the consequences of these special‐type polyps are similar to those of any conventional adenoma of this size and grade of dysplasia.

## Paneth cell metaplasia

### Synonyms

The synonyms are Paneth cell proliferation, prominent Paneth cells, Paneth cell differentiation, Paneth cell‐containing adenoma, and Paneth cell‐rich adenoma.

### Epidemiology

The prevalence of Paneth cell metaplasia in colorectal adenomas is high (range, 17–23%).^7^, ^8^, ^9^, ^10^ Paneth cell metaplasia is more frequently reported in proximal adenomas and male patients.^9^, ^11^ There is no relationship with adenoma size or the age of the patient.

### Morphology

**Figure 1:**
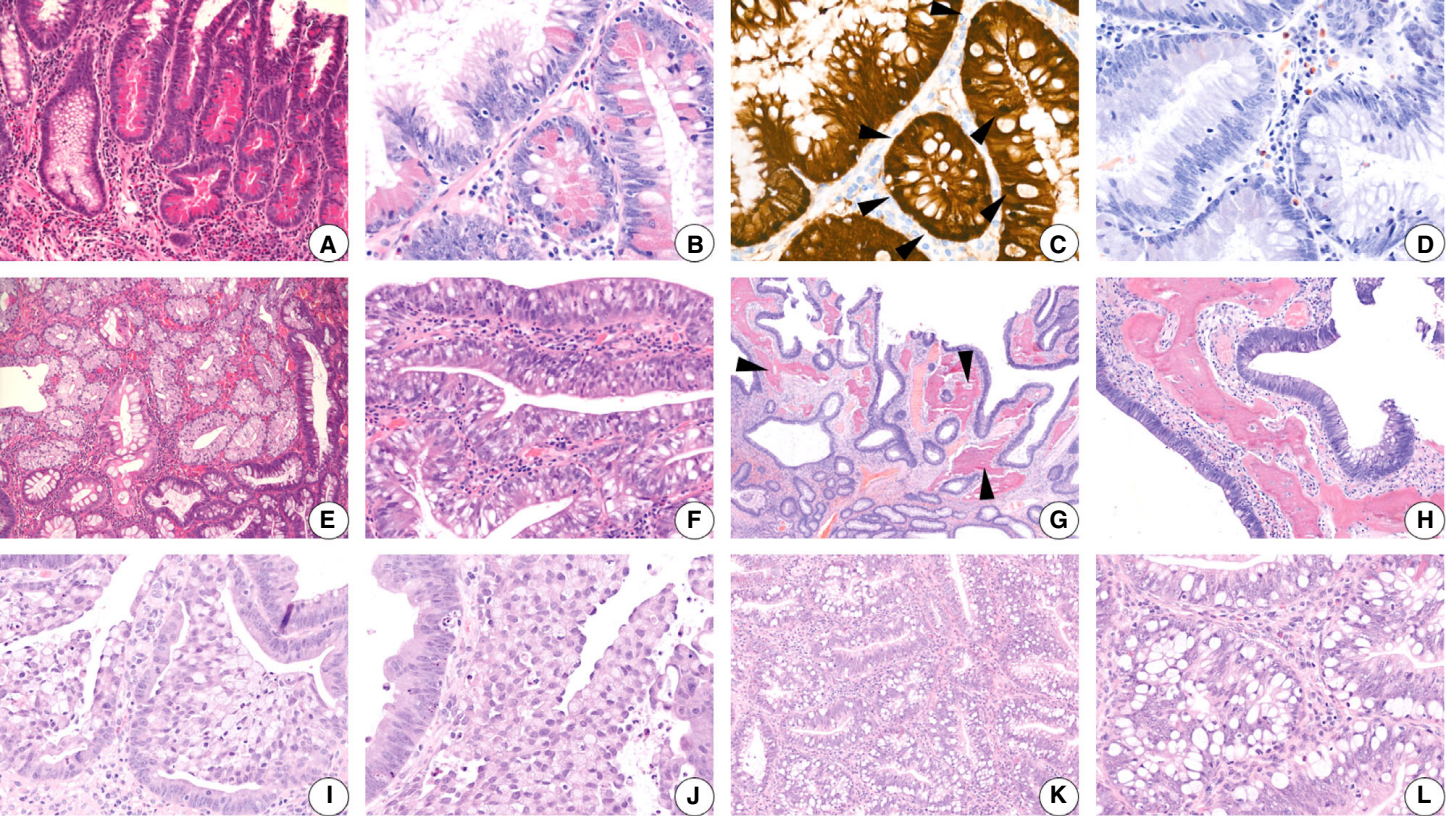
**A**–**D**, Paneth cell metaplasia (**A**,**B**, haematoxylin and eosin), with nuclear β‐catenin expression (**C**) and absence of Congo red staining (**D**).**E**,**F**, Clear cell metaplasia adjacent to dysplastic glands.**G**,**H**, Osseous metaplasia in a tubular adenoma.**I**,**J**, Signet‐ring cell‐like lesions, mainly luminal, situated in dysplastic glands.**K**,**L**, Prominent goblet cells in dysplastic glands.

Paneth cells are pyramidal cells with basally situated nuclei, and with prominent, large, eosinophilic, apical granules that occupy most of their cytoplasm.^12^ Paneth cells in adenomas show a random distribution in the adenomatous tubules, and are not confined to the basal part^9^, ^11^(Figure 1A,B).

Special stains or immunohistochemistry are not required or recommended for detecting Paneth cells, as they can be easily recognised by their distinctive morphological features. However, in the literature, various stains have been reported to facilitate research by non‐pathologists. Lysozyme can be used as a Paneth cell marker,^11^, ^13^ but lysozyme expression in gastrointestinal tissues is less specific.^11^ Congo red has been reported to enhance the visibility of Paneth cells in adenomas,^13^ but this was not the case in our study (Figure 1D). Paneth cells show uniform positivity for human defensin‐5 (HD‐5)^11^ and phospholipase A2.^14^ Non‐neoplastic Paneth cells show a normal membranous pattern of β‐catenin staining, whereas Paneth cell metaplasia in adenomas shows nuclear β‐catenin staining^11^ (Figure 1C).

### Pathophysiology

Paneth cells are normally found at the bases of the crypts of Lieberkühn in the small intestine.^12^ Paneth cells can also be present in low numbers in the proximal colon^10^ or throughout the colon as a sign of chronic inflammation in patients with inflammatory bowel disease.^15^, ^16^ The granules of Paneth cells consist of several compounds that play a role in immunity and microbial defence. Paneth cells are functionally similar to neutrophils, and release their granules into the crypt lumen via exocytosis when exposed to a variety of stimuli, including acetylcholinergic agonists and bacteria or their antigens.^12^

The mechanism of Paneth cell metaplasia in colonic adenomas remains largely unclear. Activation of the Apc–β‐catenin–Tcf signalling pathway, which is essential for intestinal homeostasis,^17^ but deregulated early in colorectal carcinogenesis,^18^, ^19^ may play a role. Nuclear accumulation of β‐catenin is a hallmark of activation of this pathway,^11^ which can be observed both in Paneth cell metaplasia adenomas in mouse models and in duodenal microadenomas of patients with familial adenomatous polyposis.^11^ This activated pathway promotes commitment towards the Paneth cell lineage via transcriptional control of specific markers of Paneth cells, such as the defensin genes.^20^ Indeed, Joo *et al*.^11^ showed that all neoplastic cells with Paneth cell differentiation showed nuclear accumulation of β‐catenin and HD‐5 expression. Because *KRAS* mutations have been identified in areas of Paneth cell metaplasia from CRC resection specimens,^7^ it has been suggested that, even without adenomatous changes, Paneth cell metaplasia can be considered to be preneoplastic.^21^ The association of Paneth cell metaplasia with chronic inflammation supports this hypothesis.^22^

### Clinical Implications

Adenomas with Paneth cell metaplasia are generally low‐grade.^11^, ^23^ Moreover, in the presence of adenomas with Paneth cell metaplasia, there seems to be a lower risk of development of synchronous or metachronous advanced adenomas or CRC.^10^ Others^9^ have suggested an increased risk for all synchronous adenomas. Adenocarcinomas with Paneth cell metaplasia are very rare.^11^, ^23^

Generally, it is believed that the presence of Paneth cells is not associated with progression or high‐risk features in adenomas.^10^, ^11^ There are no clinical implications.

## Squamous metaplasia

### Synonyms

The synonyms are squamous or squamoid morules, and squamous differentiation.

### Epidemiology

Squamous metaplasia has been observed in about 0.4% of colorectal adenomas^4^, ^24^, ^25^ (5.1% in one study^26^). There is no preferred location.^25^ The average age of the patients was 61 years (range, 39–80 years), and the majority (76%) were men.^25^ There is no information about adenoma size in relation to squamous metaplasia.

### Morphology

**Figure 2:**
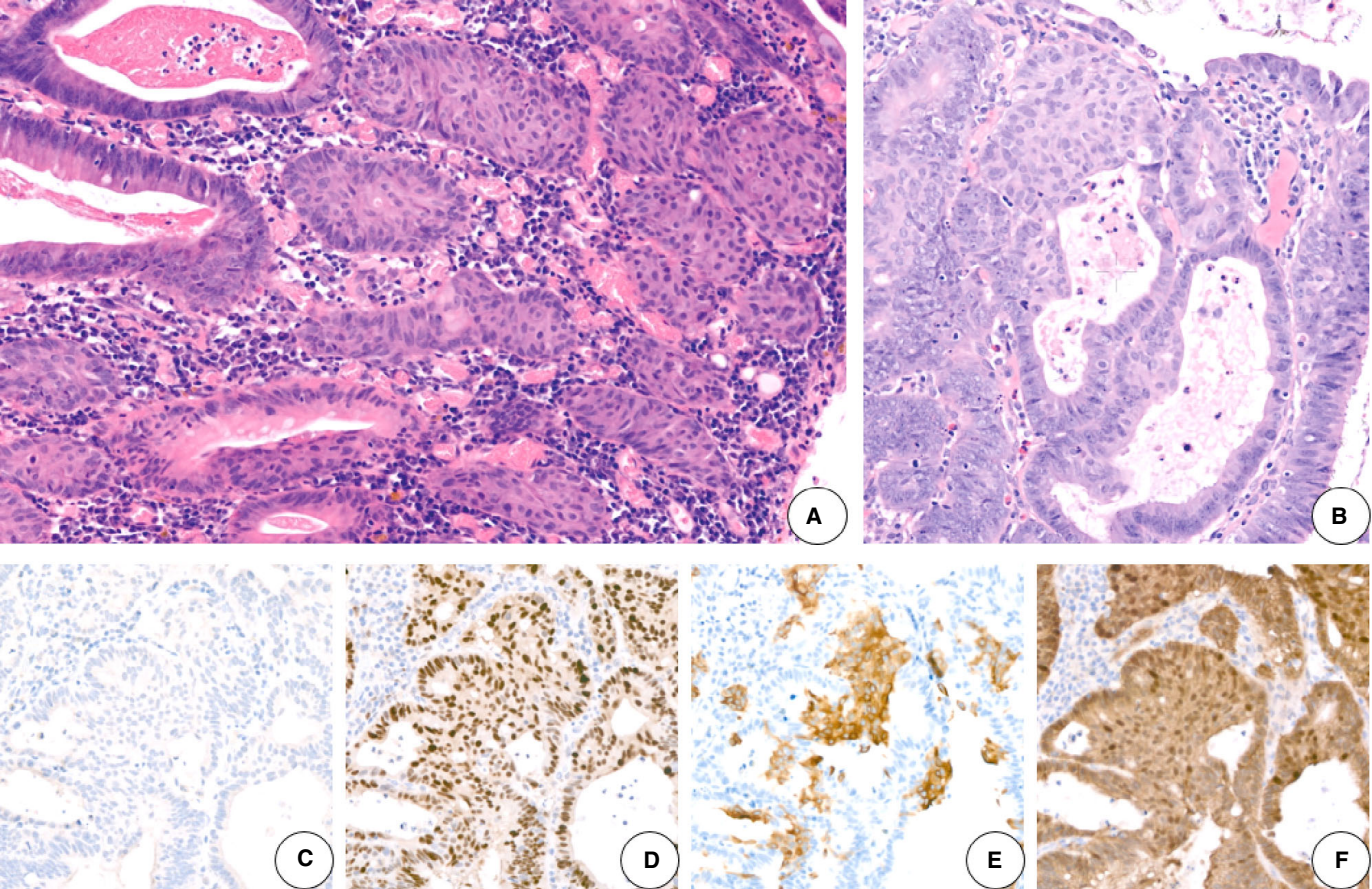
Squamous metaplasia in adenomas.**A**,**B**, Transition of dysplastic glandular structures into squamous metaplasia (haematoxylin and eosin).**C**, Absence of p63 expression.**D**, Strong cyclin D1 expression.**E**, Patchy cytokeratin 5/6 expression.**F**, Nuclear and cytoplasmic β‐catenin expression.

Squamous metaplasia in colorectal adenomas consists of nodular structures/solid nests composed of small squamous cells in association with adenomatous epithelial glands,^4^ and resembles the intraglandular morula of the endometrium^27^ (Figure 2A,B). Usually, keratin pearls, intercellular bridges or overt areas of keratinisation are not identifiable.^4^, ^13^, ^24^, ^27^, ^28^ The metaplastic epithelium is often located at the base of the adenomatous crypts.^13^, ^27^ Nodules can also protrude into the lumen of glandular structures or partially replace adenomatous glands without forming discrete nodules.^4^

Squamous metaplasia in colorectal adenomas is morphologically and qualitatively different from the conventional squamous metaplasia that is observed in other organs.^29^ Generally, colorectal squamous metaplasia cells are relatively small, round to short‐spindled, and slightly eosinophilic, with round to oval bland nuclei with clear intranuclear inclusions, so‐called ‘optically clear nuclei’; in contrast, squamous metaplasia seen in other organs is characterised by larger, polygonal, eosinophilic cells with atypical nuclei identical to those of squamous carcinoma cells without those inclusions.^29^

Pan‐cytokeratin (CK) immunoreactivity is present in areas of squamous differentiation.^13^, ^29^ CK5/6 is positive^26^ (Figure 2E) and p63 is negative within the areas of squamous metaplasia (Figure 2C); there may be focal positivity for synaptophysin and chromogranin.^4^ There is no aberrant expression of p53,^28^ but there is nuclear and cytoplasmic β‐catenin expression (Figure 2F) and cyclin D1 overexpression^26^, ^29^ (Figure 2D).

### Pathophysiology

There are several hypotheses regarding the origin of the squamous components in adenomas, including origin from heterotopic nests of squamous cells in the colonic mucosa, differentiation of the undifferentiated progenitor cell in the colonic epithelium, or metaplasia of the glandular epithelial cells into squamous cells in response to mechanical irritation, ischaemia, and chronic inflammation.^4^, ^13^, ^27^ Squamous metaplasia in colorectal polyps may even represent the precursor of primary colorectal squamous cell carcinoma.^4^, ^30^ However, primary colorectal squamous cell carcinoma outside the anal transitional zone is extremely rare.^31^

In various other neoplasms, β‐catenin mutation is involved in the histogenesis of squamous metaplasia.^28^, ^32^, ^33^

### Clinical Implications

The presence of squamous metaplasia does not affect the grading of the polyp; this should be determined in the non‐metaplastic part of the polyp. Therefore, squamous metaplasia does not alter clinical management. Because squamous metaplasia mostly presents as solid nests or pseudocribriform areas, this may be misdiagnosed as high‐grade dysplasia when the pathologist is not aware of this variant, or even mimic invasive carcinoma, especially in polyps with prolapse/torsion.^4^

## Clear cell metaplasia

### Synonyms

The synonyms are clear cell change and clear cell component.

### Epidemiology

The incidence of clear cell metaplasia in colorectal adenomas is very low; in a large series of 3486 cases, it was 0.086%.^34^ It has been suggested that these adenomas are more often observed in the distal colon and rectum,^35^, ^36^ although this could not be confirmed in other studies.^3^, ^37^, ^38^ The adenomas are not particularly large, and they are found predominantly in middle‐aged men, at an average age of 54 years.^3^, ^36^, ^37^, ^38^

### Morphology

The clear cells are predominantly columnar enterocytes,^38^ and the glands are rather compact, with scanty intervening stroma.^37^ Clear cells have clear and/or vacuolated or foamy cytoplasm. Their nuclei are predominantly pyknotic without conspicuous nucleoli or occasionally with one or more prominent nucleoli, and are usually not confined to the basal half of the cell.^3^, ^37^

The clear cells typically occupy the superficial region of the adenoma,^3^ but varying proportions of the adenoma can show clear cell metaplasia, with an abrupt transition to the remaining portion representing a typical tubular adenoma with dysplasia^37^ (Figure 1E,F).

Clear cells have no accumulation of mucin, and show no expression of Alcian blue or Periodic acid–Schiff (PAS), either before or after diastase digestion,^37^, ^38^, ^39^, ^40^ in contrast to the conventional adenomatous component.^36^, ^40^ The clear cells show an immunohistochemical pattern similar to that of the adenoma component. They usually show expression of CK20, carcinoembryonic antigen, and CDX2, consistent with the finding of a colorectal origin, and are negative for CK7, vimentin, and chromogranin.^35^, ^36^, ^37^, ^38^, ^39^

### Pathophysiology

The mechanism responsible for clear cell metaplasia is controversial and unclear; it could be an accumulation of lipid, hydropic change, or just an artefact. Histochemical and ultrastructural studies (electron microscopy) have failed to show cytoplasmic glycogen or mucin.^3^, ^38^, ^39^, ^40^ Some studies have therefore suggested a degenerative nature for clear cell metaplasia, although without any evidence.^38^, ^41^

### Clinical Implications

There are no clinical implications of clear cell metaplasia in colorectal adenomas,^3^, ^34^, ^39^, ^40^, ^42^ given the absence of clinical data.

## Osseous metaplasia

### Synonyms

The synonyms are heterotopic ossification, bony metaplasia, and heterotopic bone formation.

### Epidemiology

The incidence of osseous metaplasia is unknown, but this entity is regarded as exceedingly rare. Nine cases in total can be found in the literature.^43^

### Morphology

Osseous metaplasia refers to the formation of heterotopic bone by osteoblasts.^44^ This bone consists of irregular islands of mineralised mature osteoid bone rimmed by layers of scattered osteoblasts.^45^, ^46^ The foci of ossification in colorectal adenomas lack formation of bone marrow (Figure 1G,H). Fibrous stroma is generally present between the adenomatous component and the area of bone formation.^44^, ^47^ Bony trabeculae can be surrounded by osteoblasts. Osteocytes can be seen in lacunae with a few scattered osteoclast‐like multinucleated giant cells. There is no evidence of necrosis, cartilaginous tissue or mucin deposition in the vicinity of the bone.^44^ Sometimes, calcification can be seen. No histochemical or immunohistochemical stains are required to demonstrate bone formation.

### Pathophysiology

The cause of ossification is controversial. Associated histological features comprise inflammation, pre‐existing calcification, increased stromal vascularity, and extracellular mucin deposition.^48^ Osseous metaplasia has also been observed in rectal cancer, often after neoadjuvant treatment,^45^ and in inflammatory and juvenile polyps,^49^, ^50^ suggesting that inflammation is an important factor in ossification. In general, osseous metaplasia occurs when osteoblasts differentiate from fibroblasts secondary to persistent inflammation or tissue damage, or the release of substances such as bone morphogenetic proteins (BMPs).^46^, ^47^, ^50^, ^51^, ^52^ Local osteogenic factors such as BMPs often stimulate the osteoblasts to incorporate collagen fibres into new bone.^52^ The phenomenon can also result from the interaction of local physiochemical factors such as calcium salts (calcium phosphate) with proliferating connective tissue.^45^, ^49^, ^53^, ^54^ Most BMPs are members of the transforming growth factor‐β superfamily and play an integral role in the formation of new bone, except for BMP‐1, and hence BMP‐2, BMP‐4, BMP‐5 and BMP‐6 are present in the cytoplasm of tumour cells and within the osteoblast‐like cells of newly formed bone.^55^ Whether these factors play a role in adenomas is unknown.

### Clinical Implications

Osseous metaplasia in adenomas is generally a radiological or histological curiosity, and is an incidental finding with no prognostic or other clinical significance.^48^, ^52^

## The spectrum of neuroendocrine differentiation in adenomas

### Synonyms and Related Terms

Synonyms and related terms are neuroendocrine hyperplasia, neuroendocrine metaplasia, neuroendocrine cell proliferation, composite intestinal adenoma microcarcinoid, mixed neuroendocrine–non‐neuroendocrine neoplasm (MiNEN), and mixed adenoma–neuroendocrine tumour (MANET).

### Epidemiology

The incidence of neuroendocrine differentiation in adenomas is considered to be low (3.8% in one study),^26^ in contrast to the high percentage of CRC cases in which neuroendocrine cells have been identified. Depending on the definition^56^ and the method used to assess the neuroendocrine cell population, this varies between 8% and 77%.^56^ In an overview of MANETs, 34 cases were described, with an equal distribution between tubular and tubulovillous adenomas,^57^ and a remarkably high percentage of high‐grade dysplasia (15 of 34 cases). There were no clear associations with location and variation in size.^5^, ^57^ The patients were of a wide age range, i.e. 28–82 years, and there was a nearly equal sex ratio.^5^, ^57^

### Morphology

**Figure 3:**
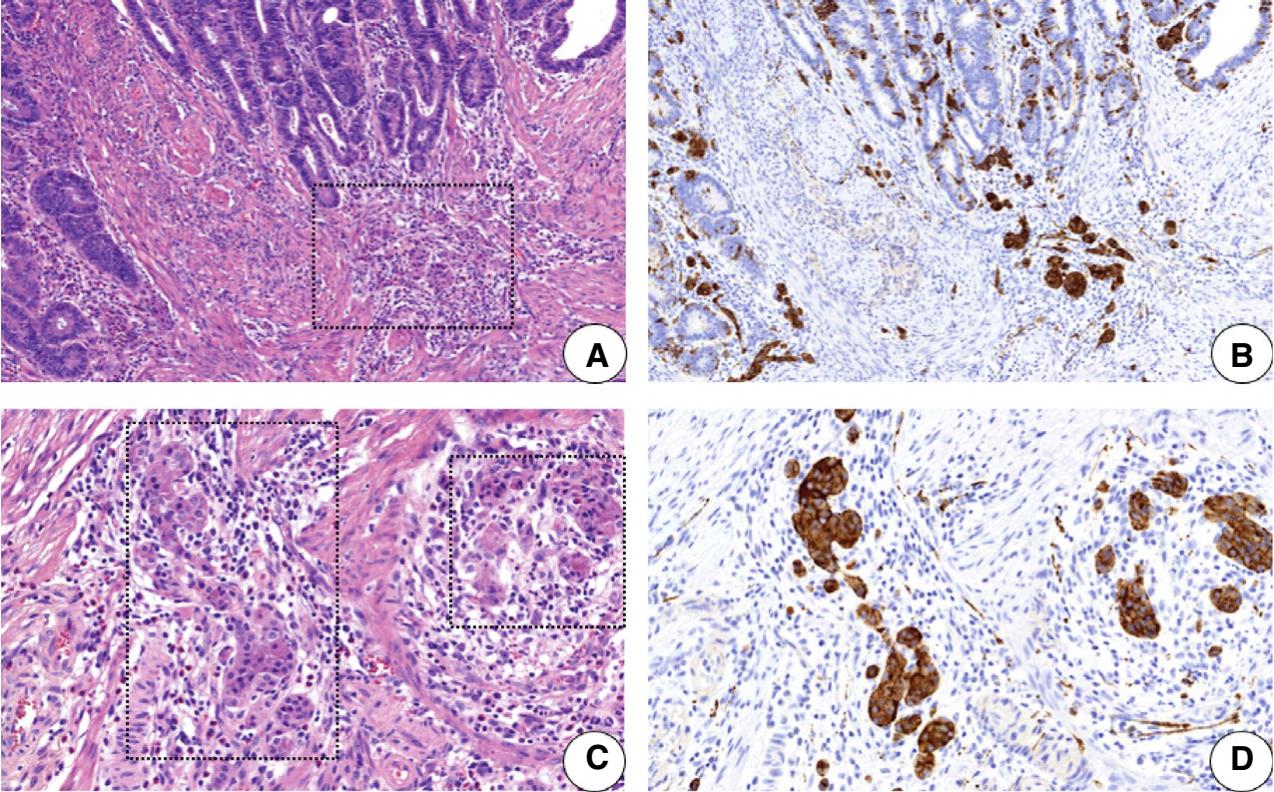
Neuroendocrine differentiation in an adenoma. There are small groups of neuroendocrine cells between and beneath the dysplastic glands (**A**,**C**, haematoxylin and eosin) with strong synaptophysin expression (**B**,**D**).

Neuroendocrine differentiation may be difficult to recognise^5^; it can be identified at high magnification as scattered foci next to and in between dysplastic glands (Figure 3A,C).^26^ Neuroendocrine foci are confirmed by immunohistochemistry for synaptophysin (Figure 3B,D) and chromogranin A.^58^ Neuroendocrine differentiation usually occupies only a minute region in the centre of the polyp, without disturbing the overall architecture or with clear demarcation from the glandular component.^5^, ^26^

The neuroendocrine component comprises <30% of the adenoma by definition.^59^ The neuroendocrine cells in adenomas are arranged in solid, gyriform, trabecular, tubuloacinar or pseudorosette patterns, with centrally located round‐to‐oval nuclei, salt and pepper chromatin, and the presence of fine eosinophilic granules on haematoxylin and eosin staining.^58^ Sometimes, budding of single neuroendocrine nests from the overlying adenomatous crypts can be seen, or even clusters of neuroendocrine nests <2 mm, limited within the lamina propria, and distributed along the base of the adenomatous crypts.^60^ They lack significant nuclear atypia, mitotic activity, or necrosis.^5^

There is no increase in the Ki67 labelling index in the cells showing neuroendocrine differentiation.^5^ Both nuclear immunoreactivity and cytoplasmic immunoreactivity for β‐catenin are usually present.^57^

### Pathophysiology

Although gastrointestinal neuroendocrine cells are of endodermal origin,^61^ neither the natural history nor the pathogenetic mechanism for neuroendocrine differentiation in adenomas has been fully elucidated, partly because of under‐recognition.^5^ Long‐standing inflammation or mechanical injury might induce chronic mucosal injury that causes neuroendocrine differentiation.^62^

Studies have proposed a role of β‐catenin,^57^, ^60^ with an altered Wnt–β‐catenin signalling pathway, and a shared pathogenesis with other special types of adenoma.^26^

### Clinical Implications

To differentiate sporadic neuroendocrine differentiation in adenomas from low‐grade MiNEN, there is a cut‐off of 30% for the neuroendocrine component.^57^, ^59^ Low‐grade MiNENs are rare, indolent lesions that constitute approximately 5% of colorectal MiNENs^59^; they usually present as polyps <30 mm in diameter, and occasionally cause carcinoid syndrome.^59^ Local excision is generally considered to be adequate.^59^

Scattered neuroendocrine cells sometimes resemble infiltrative glands or tumour budding, but there is no desmoplastic reaction.^5^ Neuroendocrine differentiation can even resemble squamous metaplasia^26^ or high‐grade dysplasia.^5^ Awareness of the possibility of neuroendocrine differentiation might thus prevent overdiagnosis. If there is doubt, immunohistochemical confirmation is helpful.

## Signet‐ring cell‐like lesions

### Synonyms and related terms

Synonyms and related terms are benign signet‐ring cell aggregates, signet‐ring cell‐like changes, non‐neoplastic signet‐ring cells, pseudo‐signet ring cells, prominent goblet cells, and goblet cell‐rich lesions.

### Epidemiology

The incidence and clinical features of signet‐ring cell‐like lesions in colorectal adenomas are not known, owing to the sparse literature. Intramucosal signet‐ring cell carcinoma is a distinct entity that is not discussed here, although it may be difficult to distinguish it from signet‐ring cell‐like lesions.

### Morphology

Signet‐ring cells are cells with characteristic crescent‐shaped and peripherally displaced nuclei.^63^, ^64^ Signet‐ring cell‐like lesions consist of sloughed goblet cells, which assume a round shape with a signet‐ring appearance.^65^ Their nuclei are not enlarged, but appear to be compressed towards the periphery of the cell. They do not show mitoses, and have a uniform chromatin distribution without atypia.^63^, ^65^ In colorectal adenomas, the formation of signet‐ring cell‐like lesions can be seen as clusters or small cell groups (Figure 1I,J), which are always confined to the surface of the mucosa or crypts of intestinal epithelium, thereby not invading/infiltrating the lamina propria.^63^, ^65^ In some cases, the crypt epithelial cells are exclusively goblet cells (Figure 1K,L) with marked goblet cell distension. These cells can be positive for PAS, PAS–diastase, and pan‐cytokeratin, similarly to true signet‐ring cells, but show a wild‐type p53 staining pattern with a low Ki67 proliferation index.^63^, ^64^, ^65^

### Pathophysiology

The formation of these cells is probably caused by stretching or torsion of the polyps, causing focal mucosal ischaemia and sloughing of the epithelium, resulting in the detachment of epithelial cells.^63^ The sloughed epithelial cells, including the goblet cells, accumulate in mucosal folds in which the surface openings are obstructed. Some of the cells may assume a round/balloon shape, and thereby a signet‐ring appearance.^65^

### Clinical Implications

The occurrence of signet‐ring cell‐like lesions in colorectal adenomas is a rare, non‐neoplastic^64^ and very misleading diagnostic pitfall.^65^ It needs to be differentiated from colorectal or metastatic signet‐ring cell carcinoma, in order to avoid unnecessary surgical resection.^63^, ^64^, ^65^

## Conclusions

Increasing recognition of conventional adenomas with special and incidental morphological features raises questions about the impact of these features. Whereas some forms of metaplasia are nothing more than diagnostic curiosities, such as Paneth cell metaplasia and osseous metaplasia, others can be diagnostically confusing.

The presence of squamous metaplasia, neuroendocrine differentiation and signet‐ring cell‐like lesions should be recognised in order to avoid overdiagnosis. Their aberrant growth pattern, which is sometimes associated with epithelial misplacement, can cause overinterpretation as high‐grade dysplasia, or even invasive carcinoma. Knowledge of the existence of these special features and the possibly helpful stains are provided in this article. Given the rarity of the majority of these features, it is not feasible to provide reliable epidemiological data. The majority of adenomas are found in middle‐aged male patients, as is the case for all conventional adenomas.

**Figure 4:**
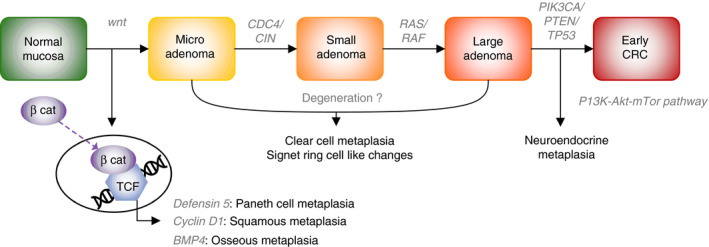
The incorporation of special features in the adenoma–carcinoma sequence: a hypothetical explanation of morphology, based on the molecular characteristics of a small series of adenomas with special features.

When the hypotheses regarding the development of these features in conventional adenomas are investigated, the Wnt signalling pathway is often involved. In the traditional descriptions of the adenoma–carcinoma sequence, this signalling pathway is the first to be disturbed, usually by mutations in *APC*.^66^ This raises the question of whether this pathway is really involved in the development of special morphological features, or whether this is just a stage in the development of any conventional adenoma. In Figure 4, we have incorporated these special features into the adenoma–carcinoma sequence. In an early stage of development, different targets of Wnt signalling can be affected, e.g. BMP‐4, which is involved in osseous metaplasia,^55^ and HD‐5 for Paneth cell metaplasia.^67^ If clear cell metaplasia and signet‐ring cell‐like lesions are caused by degeneration, there may not be a special molecular feature for those types, although this has not been studied. The development of neuroendocrine differentiation is considered to be a later event, as neuroendocrine components of CRCs share the common mutations of the non‐neuroendocrine parts, including mutations in *APC*, *TP53*, and *KRAS*.^68^, ^69^ It has been suggested that the development of neuroendocrine cells in adenomas or adenocarcinomas is influenced by the phosphoinositide 3‐kinase–Akt–mammalian target of rapamycin pathway.^70^

With the increased recognition of these features that can occur in conventional colorectal adenomas, we will learn more about their epidemiology, and these morphological findings may also give us insights into carcinogenesis pathways.

## Conflict of interests

The authors declare that no conflicts of interest exist. There was no grant support for this study.

## Author contributions

P. D. Dabir, R. S. van der Post and I. D. Nagtegaal contributed to study design and drafting of the manuscript. P. D. Dabir performed the initial literature search. R. S. van der Post and I. D. Nagtegaal reviewed the draft. All authors performed critical revision of the manuscript for important intellectual content.
